# *Pseudomonas aeruginosa* Acquisition in Cystic Fibrosis Patients in Context of Otorhinolaryngological Surgery or Dentist Attendance: Case Series and Discussion of Preventive Concepts

**DOI:** 10.1155/2015/438517

**Published:** 2015-03-18

**Authors:** Jochen G. Mainz, Andrea Gerber, Michael Lorenz, Ruth Michl, Julia Hentschel, Anika Nader, James F. Beck, Mathias W. Pletz, Andreas H. Mueller

**Affiliations:** ^1^Cystic Fibrosis Center, Pediatric Pneumology, Jena University Hospital, 07740 Jena, Germany; ^2^Center for Infectious Diseases and Infection Control, Jena University Hospital, 07740 Jena, Germany; ^3^Department of Otorhinolaryngology/Plastic Surgery, SRH Wald-Klinikum, 07548 Gera, Germany

## Abstract

*Introduction. P. aeruginosa* is the primary cause for pulmonary destruction and premature death in cystic fibrosis (CF). Therefore, prevention of airway colonization with the pathogen, ubiquitously present in water, is essential. Infection of CF patients with *P. aeruginosa* after dentist treatment was proven and dental unit waterlines were identified as source, suggesting prophylactic measures. For their almost regular sinonasal involvement, CF patients often require otorhinolaryngological (ORL) attendance. Despite some fields around ORL-procedures with comparable risk for acquisition of *P. aeruginosa*, such CF cases have not yet been reported. We present four CF patients, who primarily acquired *P. aeruginosa* around ORL surgery, and one around dentist treatment. Additionally, we discuss risks and preventive strategies for CF patients undergoing ORL-treatment. Perils include contact to pathogen-carriers in waiting rooms, instrumentation, suction, drilling, and flushing fluid, when droplets containing pathogens can be nebulized. Postsurgery mucosal damage and debridement impair sinonasal mucociliary clearance, facilitating pathogen proliferation and infestation. Therefore, sinonasal surgery and dentist treatment of CF patients without chronic *P. aeruginosa* colonization must be linked to repeated microbiological assessment. Further studies must elaborate whether all CF patients undergoing ORL-surgery require antipseudomonal prophylaxis, including nasal lavages containing antibiotics. Altogether, this underestimated risk requires structured prevention protocols.

## 1. Introduction

The autosomal recessive inherited disease cystic fibrosis (CF) remains life threatening, first and foremost because of pulmonary destruction. Pathogens like* Pseudomonas (P.) aeruginosa*, which, once established, cannot be eliminated from CF airways due to defective mucociliary clearance, remain the principal reason for premature death [1].* P. aeruginosa* and other crucial pathogens that are ubiquitously found in water, for example, in sanitary installations in the patients' surroundings [2], can be acquired by diagnostic and treatment procedures. From nebulisers, pulmonary test equipment, and other respiratory aids used in homecare and in CF centres, even* Burkholderia cepacia complex* (Bcc) has evidently been transmitted to CF patients [3]. This pathogen caused endemics within some CF centre cohorts and high mortality rates during the 1990s, before preventive measures were implemented [4]. In these times, a series of microbiological studies was performed, which revealed contamination of dental unit waterlines with* P. aeruginosa* and* B. cepacia complex* [3, 5] in up to 50% of inspected units [6]. In 1997, Jensen et al. [7] assessed water samples from different oral health care services for* P. aeruginosa* in regard to acquisition risks for CF patients. Between 2.9% and 11% of the dental sessions samples were found positive for* P. aeruginosa*. At least in one case, transmission was proven by isolation of the same genotype (identical RFLP, pulsed-field gel electrophoresis patterns) from patient and tap water.

In contrast, for otorhinolaryngological- (ORL-) surgical treatment, we did not find any reports addressing the risk for acquisition of pathogens like* P. aeruginosa*, although some procedures should be comparable to dental care. Additionally, sinonasal surgery is followed by a period of mucosal defect, when debridement and crusts further impair upper airways (UAW) mucociliary clearance and increase the likelihood of pathogen infestation, even when preventive measures have been taken both within and around surgery.

We present a single centre case series of five CF patients who acquired* P. aeruginosa* after ORL-surgery (*n* = 4) or dentist treatment (*n* = 1), performed at 4 different sites. One of the aims of the present paper is to reveal the huge heterogeneity regarding timelines of the pathogens' first detection and success rates of eradication therapy. This gives a glance on the wide spectrum of risks. Thereby, the publication shall help to identify and eliminate special contamination risks and discuss prevention strategies.

## 2. Case Reports

**Figure 1:**
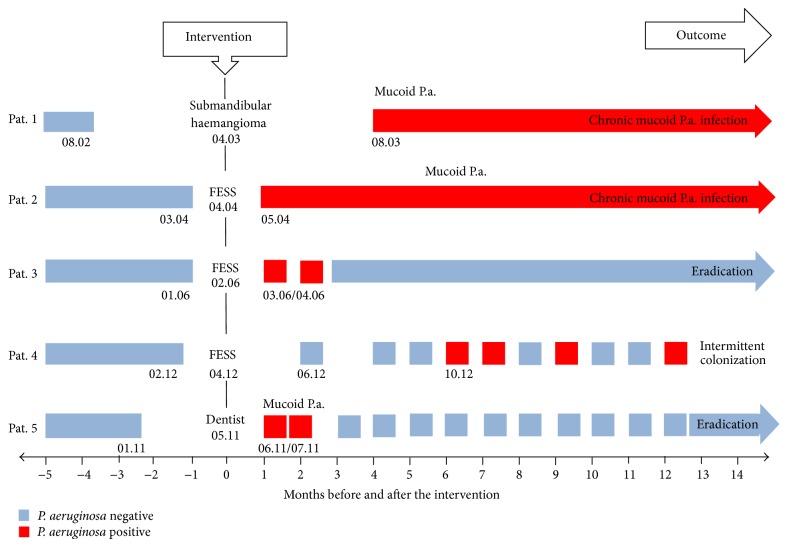
Different courses and outcomes after* P. aeruginosa* (P.a.) colonization in context of ORL-surgery or dentist treatment. Pat.: patient; FESS: functional endoscopic sinus surgery.

Graphic presentation of the five patients' course of* P. aeruginosa* colonisation in the context of the different interventions is given in Figure 1. Microbiological detection of first pathogen colonization was done within clinical routine by conventional methods in laboratories which take part in the German external quality survey [8] and following the current standards, also for differentiation of mucoid and nonmucoid phenotypes of* P. aeruginosa* [9]. Except Pat. 1 none of the five patients revealed colonization with* P. aeruginosa* before the documented interventions. Pat. 1 revealed two positive sputum cultures in 2000 which appeared to be successfully eradicated, as all cultures obtained more frequently than every 3 months resulted negative for the pathogen until he underwent surgery in April 2003.

Altogether, a learning curve is obvious: routine assessment of airway colonization implemented after our experiences with the first patients and consequent treatment of detected pathogens prompted eradication of* P. aeruginosa* in the later cases. Whereas the two early patients resulted to remain chronically colonized by mucoid* P. aeruginosa*, those receiving thorough controls and therapy evidently had better chances for eradication of the pathogen. Subsequently we present detailed information about the reported patients.

### 2.1. Patient 1, Born in 1987 (*P. aeruginosa* Acquisition around ORL-Surgery-Resection of a Submandibular Haemangioma in 2003)

**Figure 2:**
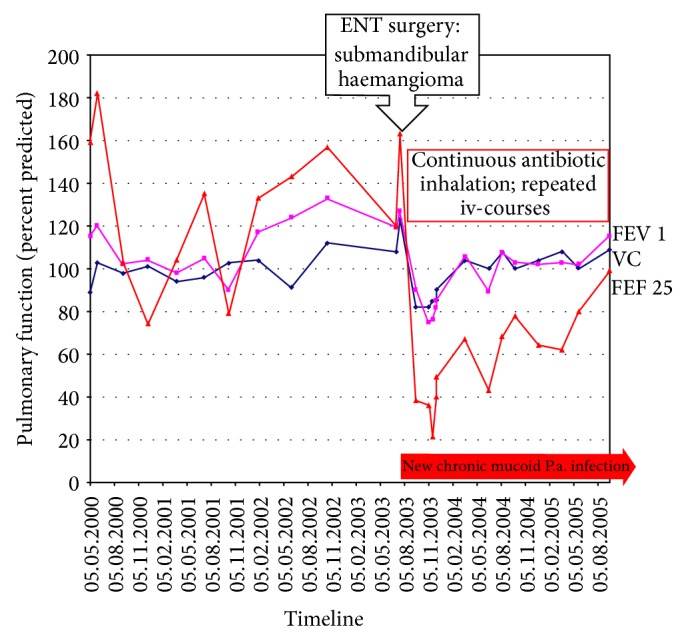
Progress of pulmonary function of patient number 1.

The male patient homozygous for F508del did not show limitations of his pulmonary function during his first 14 years of life (see Figure 2). The active soccer player's FEV1 always resulted above 100% of predicted. At the age of 15, the patient's home doctor submitted him to ORL-surgery: during intubation and mechanical ventilation a left sided submandibular tumour was resected. Histologically it was revealed to be a haemangioma with cavernous and sinusoidal vascular cavities and myxoid areas.

In contrast to the patient's usual adherence, he had not coordinated surgery with our CF centre and he did not present to our outpatient clinic for almost 4 months, despite massively increased sputum production and newly developed shortness of breath on exertion. In parallel, he lost 3 kilograms of body weight. Sputum culture then revealed mucoid* P. aeruginosa* and the previously negative serum antibodies against the pathogen resulted positive (alk. phosphatase: 1261, elastase 2.130, exotoxin 678; reference values: negative titres < 500/marginal 500–1250/positive > 1250). Pulmonal function (see Figure 2) reveals the fulminant decrease after new* P. aeruginosa* colonization, which improved considerably with daily bronchial antibiotic inhalation with tobramycin 2 × 300 mg over 28 days alternating with colomycin 2 × 1 MioIE. Additionally, he received 3-4 iv-antibiotic courses over 14 days, for example, with tobramycin and ceftazidime per year as elective therapy [10]. Nevertheless, the patient remains positive for* P. aeruginosa*. As an anti-inflammatory agent he continuously was treated with azithromycin 250 mg three times a week [10–12]. This antibiotic, which does not directly inhibit growth of pathogens like* P. aeruginosa*, has a modifying role in diseases associated with airway infection and inflammation, like CF [12].

### 2.2. Patient 2, Born in 1988 (*P. aeruginosa* Acquisition around Sinonasal Surgery in 2004)

The female patient genotype pN1303K/1343delG with mild pancreatic insufficiency was diagnosed with CF aged 12 for gastroenteral symptoms and failure to thrive. Since the age of 16, she complained about bilateral nasal obstruction progressively impairing her quality of life. Sinonasal CT scans revealed subtotal obliteration of the right nasal cavity by a large polyp and smaller polyps within the left middle nasal meatus. Additionally, both ethmoidal and maxillary sinuses were obliterated; only the left frontal sinus was developed, but totally opacified, as the sphenoidal sinus.

The patient, who had previously been treated at a different CF centre, had never revealed* P. aeruginosa* colonization. She underwent extended functional endoscopic sinus surgery (FESS) in April 2004 and received a perioperative antibiotic treatment with cefuroxime. While all lower airway samples taken before surgery resulted negative for* P. aeruginosa*, she became chronically infected: the pathogen was detected in the first microbiological control 1 month after surgery which transited quickly into a mucoid phenotype despite repeated intravenous antibiotic treatment with tobramycin and ceftazidime over 14 days and bronchial antibiotic inhalation with tobramycin 300 mg BID and colomycin 1MioIE BID. Since attendance in our centre in 2006, we additionally detected chronic sinonasal* P. aeruginosa *colonization by routine diagnostic nasal lavage (NL) (10 mL of NaCl 0.9%/nostril [13]). Since acquisition of* P. aeruginosa*, pulmonary function decreased from FEV1 103% predicted in 2004 to presently 56% in only eight years.

### 2.3. Patient 3, Born in 1990 (*P. aeruginosa* Acquisition around Sinonasal Surgery 2006)

The male patient genotype F508del/G542X was diagnosed at the age of 2 years for gastroenteral symptoms and failure to thrive. Since the age of 13 years, he suffered from chronic rhinosinusitis symptoms with impaired sense of smelling. In February 2006, at the age of 15, the patient (at that time, treated in another CF centre) underwent FESS in an external ORL department. After surgery, he reported about improved symptoms of nasal obstruction, but one month after the intervention, cough and mucus production reasoned microbiological assessment of abundant sputum. For the first time, his sputum resulted positive for a nonmucoid* P. aeruginosa*, a finding confirmed in the control after 3 weeks.

**Figure 3:**
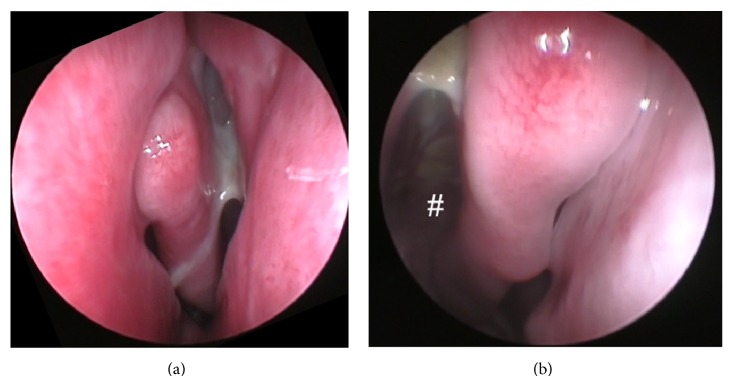
(a) and (b) Anterior rhinoscopy from patient number 3 six years after sinonasal surgery. (a) Abundant mucoid secretions draining from middle meatus left side; (b) # view into postoperative polyp-free ethmoid sinus, mucoid secretion on the roof of the sinus.

The patient underwent a 2-week iv-course with tobramycin and ceftazidime and a 6-month course of bronchial inhalation with colistin. Since then, we did not find* P. aeruginosa* in routine upper and lower airway sampling, but the patient soon again started to suffer from nasal obstruction and postnasal drip with abundant mucoid secretions from his paranasal sinuses (Figures 3(a) and 3(b)).

### 2.4. Patient 4, Born in 2003 (*P. aeruginosa* Acquisition Months after Sinonasal Surgery 2012)

The female patient genotype F508del/W1282X was diagnosed with failure to thrive at the age of 3 months. With substitution of pancreatic enzymes, she gained weight but always remained thin and revealed chronic rhinosinusitis symptoms with severe nasal obstruction. At the age of 7 years, adenoidectomy was performed in an external ORL-clinic, but her preexisting early nasal polyps remained untouched. Soon after surgery, sinonasal symptoms reoccurred with massive nasal obstruction and severely impaired senses of smelling that contributed to her continuously poor nutritional status.

**Figure 4:**
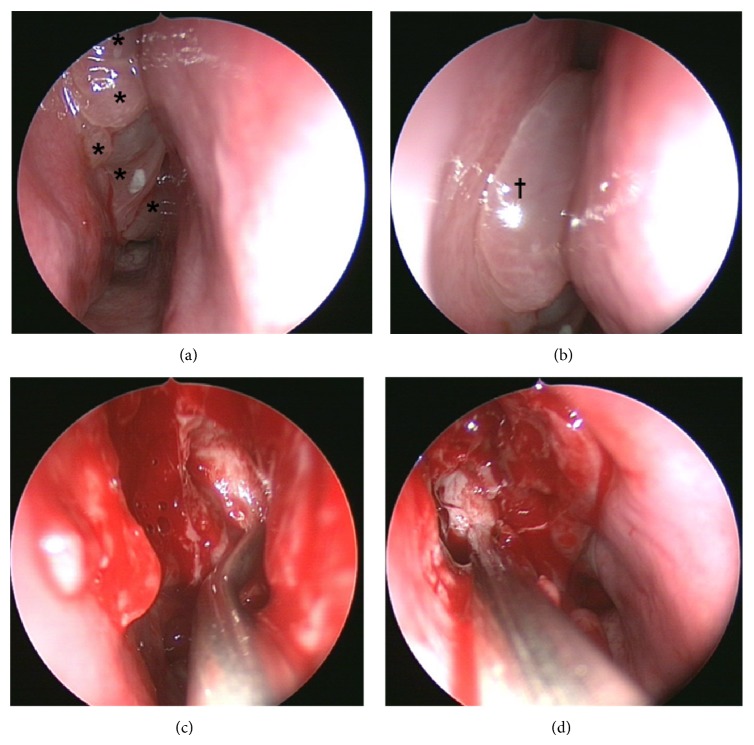
(a) and (b) right and left nasal sides prior to sinonasal surgery with a series of apical polyps (∗) on the right (a) and a big floating polyp (†) on the left nasal side, besides the middle turbinate. (c) and (d) Suction tube inside the opened left (c) and right (d) maxillary sinus.

In April 2012 (at the age of 9 yrs), the patient underwent MRT/CT-guided FESS in a second ORL-clinic, with resection of nasal polyps and hypertrophic mucosa, and widening of sinus ostia (Figures 4(a)–4(d)). During postoperative care, she was treated with nasal lavages with isotonic saline (250 mL) and topical steroids but no antipseudomonal antibiotics. She reported massive improvement of olfactory function after surgery, but, at the same time, the family complained about severely smelling nasal crusts.

She was monitored very closely for upper and lower airway pathogen colonization by sputum and diagnostic NL (10 mL/nostril). Remarkably, we did not detect* P. aeruginosa* airway colonization for the 5 postoperative months in deep throat swabs or NL. Then, in October 2012, during enhanced sputum production for acute airway exacerbation, lower airway swabs revealed a first new nonmucoid* P. aeruginosa* colonization, confirmed in early November 2012. The patient underwent a 2-week antipseudomonal iv-course and additionally a 6-week oral treatment with ciprofloxacin. She inhales colistin 2x1MioIU bronchially, in addition to colistin 2x1MioIU sinonasally as vibrating aerosols (Pari-Sinus) [14, 15]. Since the antipseudomonal treatment, stinking nasal crusts dissolved, but the pathogen was again detected in her lower airways.

### 2.5. Patient 5, Born in 1979 (*P. aeruginosa* Acquisition within Dentist Care in 2011)

This female patient, genotype R347P/2183AA->G, had always been free of* P. aeruginosa* in frequent upper and lower airway cultures. However, she has been suffering from chronic rhinosinusitis for many years, prompting ORL-surgery in October 2002 and January 2006 without complications but with early relapse of sinonasal symptoms.

In May 2011, the 32-year-old patient underwent dental treatment that included drilling at a molar tooth. Few weeks afterwards, she reported increased sputum production and cough. According to the standards of care established in our CF centre, sputum and NL fluid were obtained, collected as previously described [16]. Remarkably, mucoid* P. aeruginosa* was detected in her sputum, but NL only revealed* Staphylococcus (S.) aureus* 100.000 cfu/mL. The colonization status was confirmed by microbiological controls before starting a 2-week antipseudomonal iv-course with ceftazidime and tobramycin, in addition to bronchial and sinonasal inhalation with colistin and oral azithromycin.

## 3. Discussion

Aquatic biofilms that contain microbial communities of* P. aeruginosa*,* B. cepacia complex*, and other pathogens are ubiquitous in nature, frequently found in water lines, and therefore also widespread within medical and dental devices [3, 6]. In such locations, especially, pathogens with intrinsic resistance to biocides and high temperature resistance may diversify and spread. For susceptible hosts, they can cause serious infections.

In patients with the inherited disease CF, viscous airway secretions impair mucociliary clearance, causing these organisms to not be properly eliminated from the airways. Pathogens like* P. aeruginosa* damage the airways not only by their virulence factors but also by causing enhanced and frustrated host immune responses leading to pulmonary destruction, the primary reason for premature death in CF [1].

Therefore, identification and elimination of sources with risk to spread pathogens into CF airways is of primary importance. Moreover, as members of the care health system, we owe highest responsibility to our patients to prevent nosocomial infections in those consulting us and to prevent risks that may follow our treatment. Thus, we feel responsible for sharing and discussing the course of 5 CF patients and for calling for concepts to prevent repetition.

The presented spectrum is of CF patients with diverse history, now attended in a single centre, who underwent otorhinological surgery or dentist treatment in four different locations. All of them appear to have acquired* P. aeruginosa* in the course of the surgical intervention or in evident time relation after it.

With our case series we want to sensitize CF care takers and the cooperating otorhinological surgeons or dentists of the special risk for patients with impaired airway clearance or immunodeficiency. We call for interdisciplinary concepts of prevention of* P. aeruginosa* acquisition during procedures frequently conducted in CF patients.

### 3.1. ORL-Treatment

Within the last years, sinonasal involvement in CF is coming into the clinical and scientific focus [14, 16–24]. Almost one-third of CF patients have symptoms that fulfil the “European Position Paper on Rhinosinusitis and Nasal Polyps” (EPOS) [25] criteria of chronic rhinosinusitis, and another third suffers from remittent symptoms [26]. Thereby, almost all CF patients reveal pathologic sinonasal CT scans [27]. In consequence, routine ORL-care, at least once a year, has been postulated for every CF patient [19], and for many of them therapeutic decisions must be taken. However, the paranasal sinuses, which are frequently obliterated by inflamed mucosa, viscous mucus, and polyps, have been recognized as a site of first and persistent colonization of CF airways with pathogens like* P. aeruginosa* [14, 16, 23, 28]. Regardless, sinonasal sampling for detection of colonizing pathogens does not yet belong to the current standards of CF care.

When ORL-surgery is required, structured preventive measures should be taken.

### 3.2. Impact of Our Case Report Collection on ORL-Treatment of CF Patients

Until now, no standards of perioperative care for patients with CF in head and neck surgery, including oral and maxillofacial surgery, have been established in Germany nor in many other countries.

This includes the following aspects:

- identification of risks within the following:
  - presurgical attendance (including segregation from pathogen carriers and diagnostic procedures at risk of pathogen transmission),
  - surgery (including instrumentation, drilling, and flushing),
  - anesthesia (intubation, mechanical ventilation, suction catheters, reusable laryngeal masks, and multiple used ventilation hoses),
  - postoperative treatment (including routine procedures, such as inhalation on ward, nasal clearance, and segregation from pathogen carriers);
- diagnostic programs for detection of new pathogen colonization;
- antibiotic prophylaxis and adjuvant therapy;
- antibiotic treatment in case of pathogen acquisition.

Around dentist treatment (see Introduction), pathogens like* P. aeruginosa *and* B. cepacia complex *are well recognized as nosocomially relevant, as they persist within dental waterlines from where they can be nebulized into CF airways, for example, during drilling and flushing procedures [5–7].

In distinction to dental treatments, ORL-surgery with pre- and postoperative controls in outpatient clinics, anesthesia with mechanical ventilation, and in-hospital treatment around surgery are associated with a wide range of risks. For example, about 36% of patients with external otitis are reported to be colonized by* P. aeruginosa* [29], which also is frequent in patients with tracheostomy. By direct contact in waiting areas and indirectly by surfaces and personnel attending the patients, such pathogens can be transferred. We expect highest risks for close succession of instrumental treatment of patients with various ORL diseases (i.e., FESS patients with CF treated along with other patients on the same treatment course/center). The same applies for treatment in ORL outpatient clinics after discharge from hospital.

### 3.3. Impaired Mucociliary Clearance Consecutive to Sinonasal Surgery

Patient number 3 in our case series may give an underestimated reason for consistent controls: the patient revealed first* P. aeruginosa* detection 6 months after surgery. This patient may elucidate the important role of impaired mucociliary clearance consecutive to surgery.

Already in 1948, Hilding [30] showed in a canine model that sinonasal surgery is associated with scar formation and disruption of mucociliary clearance with debridement and resection of mucosa. In humans, a more recent study proved that sinonasal surgery is followed by impaired ciliary beat frequency (assessed by microscopic photometry) requiring up to 6 months to reach normal values in non-CF patients [31]. Besides, FESS creates a wound area in the middle meatus and the ethmoid sinus with exposed bone and epithelial defects. Reepithelialisation takes 6–12 weeks while being an ideal adhesion surface for bacterial biofilms and crusts. Even in non-CF patients, antibiotics requiring infections, including sinusitis, are not uncommon in this post-FESS situation.

We expect the problem to be even more pronounced in CF patients, as the mucosal CF-transport regulator (CFTR) defect persists after surgery and persistently thick mucoid secretions impair sinonasal mucociliary clearance.

In some centres, an increasing proportion of patients undergo sinonasal surgery to extract obliterating nasal polyps, mucoceles, and inflamed thickened mucosa, as well as for drainage of secretions from the sinuses by enlargement of paranasal sinus ostia [24, 32]. Approaches to operate UAW routinely in CF cohorts arose within lung transplantation (LTX) programs in the early 1990s [33–35] and were expanded to wide indications. For example, within a Copenhagen/Denmark interdisciplinary study program for sinonasal surgery in CF [22, 36], extended FESS is applied for one of three defined inclusion criteria as follows:

1. search for an infectious focus in patients with increasing frequency of positive lower airway cultures or repeatedly declining lung function >10% despite intensive antibiotic chemotherapy, patients with an unknown infectious focus and increasing antibodies against* P. aeruginosa*,* A. xylosoxidans*, or* B. cepacia complex*;
2. LTX within the preceding year;
3. severe rhinosinusitis symptoms according to the EPOS [25] guidelines.

Like other ORL-programs in CF [33–35, 37], this concept includes serial antimicrobial lavages after maximal widening of the maxillary antrum within FESS. In 1995, Davidson et al. [34] from San Diego published a prophylactic protocol for rhinosinusitis surgery in CF patients undergoing LTX. FESS was followed by NL containing antipseudomonal antibiotics like colistin or tobramycin for the extremely high relapse rates of rhinosinusitis after sinonasal surgery in CF patients. In these programs, a large middle meatal maxillary antrostomy is performed, such that flushed lavage fluid reaches also the maxillary sinuses. In some centres, application was directed into the maxillary sinuses by insertion of irrigating catheters [34] (e.g., 250 mL of NL with 20 mg of tobramycin added to the last 50 mL). However, subsequent protocols dropped the cannulation concept for the easier and less invasive flushing of the nasal cavity and the assessable paranasal sinuses from a nasally applied flask.

In the same year, Moss and King [33] published a retrospective case series comparing 32 CF patients who had received FESS with additional serial antimicrobial lavages to 19 CF patients who had undergone only FESS. Relapse rates requiring repeated surgery were impressively lower in the patients with additionally serial antibiotic lavages (after 1 year 10% versus 47%, and after 2 years 22% versus 72%).

Very recently, Vital et al. [24] (Zürich, Switzerland) published results from their LTX-program. Between 1992 and 2009, 82 of 92 CF patients receiving LTX underwent posttransplant FESS and daily nasal douching to which different antibiotics were added according to microbiological findings. The authors report to have eradicated* P. aeruginosa* from more than one-third of the 77 patients (79%) harbouring the pathogen in the UAW.

In Copenhagen, CF patients with FESS additionally undergo a program of conservative antibacterial and anti-inflammatory treatment. It includes a two-week intravenous antibiotic treatment, daily NL with 250 mL of isotonic saline and addition of 3 MioIU of colistin (1/2 yr), and nasal topical steroids (1 yr) [36]. Within ORL follow-up visits, the UAW is cleansed from debridement and crusts, and secretions are taken by suction/swabs for microbiological assessment.

However, besides centres which primarily rely on surgical therapy [22, 24], the still most common approach is to regard conservative medical management as the initial step in treating sinonasal disease in CF [17]. Therefore, evaluation of conservative approaches which may reduce or postpone the need for surgery and reduce relapse rates [38] is required. In this regard, we investigated sinonasal vibrating inhalation with mucolytics and antibiotics in prospective controlled trials [15, 39]. Additionally, we apply topical steroids, even in small CF children with nasal obstruction, as well as nasal lavages for cleansing of mucus and crusts.

### 3.4. Upper Airway Sampling after Sinonasal Surgery and Dentist Treatment

Our case reports call for a structured program of upper and lower airway sampling before and after sinonasal surgery and dentist treatment.

We would advocate easy and transferable methods, applicable within routine care. In comparison to nasal blowing samples and deep nasal swabs, we previously identified diagnostic NL with 10 mL of isotonic saline per nostril as methods of noninvasive UAW-sampling for detection of sinonasal colonization with* P. aeruginosa* [16]. While blowing samples detected the pathogen in only 10% of patients chronically colonized in the lungs, 28.9% were positive in deep nasal swabs and 55.6% in NL. For more than 9 years now, we have included sinonasal sampling by NL into our routine CF microbiology program, additional to sputum sampling.

In regard to our case reports and the high chances to eradicate* P. aeruginosa* if detected early, we would propose monthly NL and sputum controls at least for the first half year following surgery.

### 3.5. In Consequence of Our Reported Cases, the Following Preventive Options around Otorhinological Outpatient Treatment and Surgery Are Recommended

1. CF patients in outpatient clinics and operating rooms should be moved to programme point 1 to exclude contamination by previous patients. Furthermore, a new workplace has to be prepared.
2. The usual perioperative antibiotic prophylaxis in FESS does not cover pathogens like* P. aeruginosa* and should be adjusted for patients with CF and continued in the postoperative course of treatment.
3. Later in the postoperative nasal care (and preoperatively) performed by hospital and practice physician, CF patients should be seen at the beginning of consultation and sterile disposable suction and sterile nasal specula have to be used.
4. Antibiotic additives used for antipseudomonal therapy or in case of LTX could be used prophylactically for postoperative NL in the first 3–6 months (until recovering of mucosal barrier).

Further on, we need evidence whether iv-antibiotic courses may be required for every* P. aeruginosa* negative patient requiring sinonasal surgery. An additional interesting question for further investigations is whether azithromycin could help to improve the course of CF patients after sinonasal surgery [12]. Possibly, its anti-inflammatory potential could improve wound healing and reduce relapse rates after ENT surgery. Furthermore, azithromycin was shown to reduce adhesion of* P. aeruginosa* to surfaces and it appears to prevent early biofilm formation in CF patients [40].

Shortcomings of the present paper are attributed to its' character of a case series. This is not a prospective study assessing a cohort of patients over years but a retrospective presentation of five patients that we consider as relevant, as acquisition of* P. aeruginosa* around ENT surgery has not been reported previously, to our knowledge. We suppose that most sinonasal interventions in CF patients are not followed by new airway colonization with the pathogen. However, as evident for dentist treatment [3, 5, 6], our case series highlights potential risks around ENT surgery in patients with impaired mucociliary clearance. Additionally it underlines that further prospective trials are required in this field including programs to monitor sinonasal and bronchial colonization prior to and after sinonasal surgery and programs assessing antibacterial therapy, which together are essential in reducing risks of pathogen acquisition around surgical procedures.

## 4. Conclusion

Our case series is proposed to bring attention to the risk of* P. aeruginosa* acquisition within ORL-care of CF patients, just as within dental care. We want to encourage development of interdisciplinary guidelines between CF specialists, ORL, and dental surgeons as well as microbiological and hygiene specialists to prevent risks to acquire pathogens for susceptible patients.
